# Combined transcriptome and metabolome analysis reveals the regulatory network of histidine kinase QseC in the two-component system of *Glaesserella parasuis*

**DOI:** 10.3389/fmicb.2025.1637383

**Published:** 2025-08-29

**Authors:** Xuefeng Yan, Yuhong Zhou, Songwei Liu, Congwei Gu, Wudian Xiao, Mingde Zhao, Zehui Yu, Lvqin He

**Affiliations:** ^1^School of Physical Education, Southwest Medical University, Luzhou, China; ^2^School of Pharmacy, Southwest Medical University, Luzhou, China; ^3^Department of Technology, Experimental Animal Center, Southwest Medical University, Luzhou, China; ^4^Model Animal and Human Disease Research of Luzhou Key Laboratory, Southwest Medical University, Luzhou, China

**Keywords:** *G. parasuis*, two-component system, *qseC*, transcriptomics, metabolomics, bacterial pathogenesis

## Abstract

**Introduction:**

*Glaesserella parasuis* (*G. parasuis*) causes agent Glässer’s disease in swine. This study investigated the mechanism of QseC in *G. parasuis*.

**Methods:**

The study utilized transcriptomic and metabolomic sequencing techniques. The Δ*qseC* mutant was examined using transmission electron microscopy.

**Results:**

Transmission electron microscopy revealed that Δ*qseC* mutant exhibited cell wall dissolution and cytoplasmic rarefaction, indicating membrane homeostasis disruption. Metabolomics analysis identified 819 metabolites, with 24/36 showing significant alterations in positive/negative ion modes. KEGG enrichment indicated abnormalities in amino acid synthesis and quorum sensing. Transcriptomic revealed 663 differentially expressed genes (DEGs), including upregulated membrane synthesis genes (*plsB* and *wecA*) and downregulated virulence factors (*hrpA* and *pilW*). Integrated analysis demonstrated that *plsB* and *wecA* formed association networks with methionine and prostaglandin metabolites.

**Discussion:**

These results establish QseC’s global regulatory role in *G. parasuis*, providing insights for novel control strategies.

## 1 Introduction

*Glaesserella parasuis* is a Gram-negative bacterium of the Pasteurellaceae family, exhibiting pleomorphic morphology (spherical, rod-shaped, and filamentous forms) and non-motility (Neil et al., 1969). As an NAD-dependent pathogens, it colonizes porcine respiratory tracts and causes polyserositis under stress conditions (Segalés et al., 1997). Affected pigs display characteristic clinical manifestations including fibrinous polyserositis (peritonitis and“fuzzy heart”), arthritis, meningitis, and sepsis (Neil et al., 1969; Castilla et al., 2012). The distinct clinical presentations of acute versus chronic infections likely reflect strain-dependent virulence factors and host immune variability (Oliveira and Pijoan, 2004). Despite its significant pathogenicity, the molecular mechanisms of *G. parasuis* infection remain poorly characterized, and no effective broad-spectrum vaccines currently exist-presenting substantial challenges for disease control.

The histidine kinase QseC, a conserved membrane-bound sensor in the QseB/QseC two-component system (TCS) of *G. parasuis* (Sperandio et al., 2002; Rooks et al., 2017). TCS primarily comprises histidine protein kinase (HPK) on the cell membrane and response regulator (RR) in the cytoplasm (Stock et al., 2000). In *Escherichia coli*, *qseC* deletion disrupts QseB dephosphorylation, establishing a dysregulated positive feedback loop that attenuates virulence gene expression (Hadjifrangiskou et al., 2011). Given QseC’s established role as a virulence amplifier in diverse pathogens (Rasko et al., 2008) (including diffusely adhering *E. coli* O104: H4 and enteropathogenic *E. coli*) (Curtis et al., 2014), and its involvement in regulating antibiotic resistance via crosstalk with PmrAB TCS (Fernandez-Ciruelos et al., 2023), this system represents a promising target for novel antibacterials.

The *qseC* gene is highly conserved across *G. parasuis* serotypes serotypes 1–15 (Yan et al., 2022). We previously constructed a Δ*qseC* mutant in serotype 13 and demonstrated that deletion: (i) reduces tolerance to osmotic/oxidative/thermal stress, (ii) impairs iron acquisition and biofilm formation, and (iii) attenuates virulence in murine models (He et al., 2018). Notably, recombinant QseC protein activates cellular immunity in mice and triggers pro-inflammatory cytokine secretion (IL-1β, IL-6, TNF-*α*) in RAW 264.7 macrophages via JNK MAPK, p38, and NF-κB pathways (Yan et al., 2024). While QseB phosphorylation is directly controlled by QseC kinase activity, the mechanistic details of their interaction remain unresolved. To address this gap, we performed comparative transcriptomics of wild-type MY1902 and Δ*qseC* strains.

Here, we aimed to elucidate the global regulatory network governed by QseC in *G. parasuis* through integrated RNA-seq and UHPLC-Q-TOF MS-based metabolomics. By synthesizing transcriptional and metabolic profiles, this study specifically seeks to: (i) identify molecular mechanisms underlying the Δ*qseC* phenotypic alterations, and (ii) establish a multi-omics framework for understanding QseB/QseC-mediated pathogenesis in *G. parasuis*.

## 2 Materials and methods

### 2.1 Bacterial strains and culture conditions

The wild-type *G. parasuis* strain MY1902 (serotype 13) was generously provided by the Swine Disease Research Center at Sichuan Agricultural University. The Δ*qseC* mutant and its complemented strain (c-Δ*qseC*) were constructed via homologous recombination as detailed in previous study (He et al., 2018), with all strains cryopreserved at −80 °C in our laboratory repository. The Δ*qseC* mutant was constructed by replacing the gene with a kanamycin resistance cassette using overlap PCR and homologous recombination via the suicide vector pK18*mobsacB*. The complementation strain was generated by cloning the *qseC* gene into the replicative vector pSF116 and introducing it into the mutant. Strains were cultured Tryptic Soy Broth/Agar (TSB/TSA; BD-Difco) supplemented with 5% heat-inactivated newborn bovine serum (Solarbio) and 0.1% (w/v) NAD (Sigma-Aldrich). Kanamycin (100 μg/mL) was added for mutant selection.

### 2.2 Observation of the internal structure of bacteria

The effect of *qseC* gene deletion on the morphology of *G. parasuis* was assessed using TEM. The wild-type strain MY1902 and Δ*qseC* mutant strain were inoculated into fresh TSB at a 1:100 dilution and cultured at 80 rpm for 24 h. When the OD_600 nm_ reached approximately 1.2, the culture was centrifuged at 4,000 rpm for 10 min. The resulting bacterial pellet was gently rinsed once with phosphate-buffered saline (PBS) and re-centrifuged at 5,000 rpm for 2 min. After discarding the PBS supernatant, we added 3 mL of electron microscopy fixative (2.5% glutaraldehyde solution) to the pellet. The pellet was then gently resuspended and dispersed in the fixative. Fixation was performed at room temperature in the dark for 2 h, followed by storage at 4 °C. The fixed samples were subsequently processed and imaged using a transmission electron microscope by a commercial service provider. Images were acquired and analyzed to evaluate morphological differences.

### 2.3 Metabolite extraction for LC–MS/MS analysis

Intracellular metabolites were extracted from exponentially growing cultures (OD600 ≈ 0.8) of both the *G. parasuis* MY1902 and the Δ*qseC* mutant strain to compare their metabolite profiles. Cells were harvested by centrifugation (4 °C, 4,000 rpm, 5 min) from TSB cultures. A representative aliquot of the resulting bacterial pellet was subjected to metabolite extraction. For this purpose, the pellet aliquot was resuspended in 300 μL of ice-cold 80% (v/v) aqueous methanol to quench metabolism and initiate extraction. Immediate flash-freezing in liquid nitrogen for 5 min was performed to further arrest metabolic activity and facilitate cell disruption. The frozen suspension was then thawed on ice and vortexed vigorously for 30 s. Complete cell lysis was achieved by ultrasonication for 6 min on ice to ensure efficient metabolite release. Cellular debris was removed by centrifugation (4 °C, 5,000 rpm, 1 min). The metabolite-containing supernatant was carefully transferred to a new tube and lyophilized to dryness to concentrate the metabolites and remove volatile solvents. The dried metabolite extract was subsequently reconstituted in a volume of 10% (v/v) methanol solution equivalent to the initial pellet aliquot volume to achieve a consistent concentration suitable for LC–MS/MS injection. Finally, the reconstituted extracts were analyzed by liquid chromatography–tandem mass spectrometry (LC–MS/MS) to characterize the metabolite composition.

### 2.4 Data preprocessing and metabolite identification

Raw mass spectrometry data files (.raw format) acquired from LC–MS/MS analyses were imported into Compound Discoverer (CD) software (version 3.3) for comprehensive preprocessing. Initial data processing included alignment of chromatographic retention times and mass-to-charge ratios (m/z) across all samples to ensure consistent feature detection and matching. To correct for potential instrumental drift and enhance quantitative accuracy, peak areas were normalized relative to the first quality control (QC1) sample injected during the analytical sequence. Putative metabolite identification was then performed by matching the experimentally observed molecular ion masses ([M + H]^+^, [M−H]^−^, etc.) and characteristic fragmentation patterns against spectral libraries within the mzCloud, mzVault, and Masslist databases. Signals originating from solvents, reagents, or background contaminants were removed by subtracting features consistently detected in blank (solvent-only) samples run alongside the biological samples. For relative quantification, the raw peak area of each metabolite in each sample was normalized using the following formula to account for variations in overall metabolite abundance and instrumental response: Relative Peak Area = (Raw Peak Area of Metabolite in Sample)/(Total Sum of All Metabolite Peak Areas in Sample/Total Sum of All Metabolite Peak Areas in QC1). This normalization yielded the relative abundance of each metabolite. To ensure data quality and reproducibility, metabolites exhibiting a coefficient of variation (CV) of relative peak area exceeding 30% across the replicate QC samples were excluded from further analysis. The final curated dataset comprised the confidently identified metabolites and their corresponding relative quantitative values, providing the basis for subsequent statistical comparisons between strains. Processed metabolomics data underwent multivariate analysis using the metaX software package, where log-transformed and scaled data were subjected to Partial Least Squares-Discriminant Analysis (PLS-DA) to derive the Variable Importance in Projection (VIP) score for each metabolite. For univariate analysis, Student’s *t*-test was applied to calculate the statistical significance (*p*-value) of intergroup differences for individual metabolites, while the fold change (FC) between groups was computed to quantify relative abundance differences. Differentially abundant metabolites (DAMs) were defined using the following thresholds: VIP > 1.0, FC > 1.2 or FC < 0.833, and *p* < 0.05.

### 2.5 Statistical analysis of metabolome

Identified metabolites were functionally annotated using the Kyoto Encyclopedia of Genes and Genomes (KEGG), Human Metabolome Database (HMDB), and LIPID MAPS databases to assign putative biological roles and pathway associations. For multivariate analysis, the processed metabolomics data were log-transformed (or otherwise scaled) and mean-centered using the metaX software package prior to dimensionality reduction. Principal Component Analysis (PCA) was performed to visualize inherent data structure and detect potential outliers. Partial Least Squares-Discriminant Analysis (PLS-DA) was subsequently applied to maximize the separation between the MY1902 parental strain and the Δ*qseC* mutant groups and to identify metabolites contributing most significantly to this discrimination. Differentially abundant metabolites (DAMs) between the two strains were selected based on the following combined criteria: a Variable Importance in Projection (VIP) score > 1 from the PLS-DA model, a *p* < 0.05 derived from univariate statistical testing (e.g., Student’s *t*-test or Wilcoxon rank-sum test), and an absolute fold change (FC) ≥ 2. Pairwise Pearson correlation coefficients among the DAMs were calculated using the cor () function in R. The statistical significance of these correlations was assessed using the cor.mtest () function in R, with a *p* < 0.05 considered significant. To elucidate the biological implications of the observed metabolic alterations, functional enrichment analysis of the DAMs was conducted based on KEGG pathways. A pathway was considered enriched if the proportion of DAMs mapping to it (x/n) significantly exceeded the proportion of all identified metabolites mapping to the same pathway (y/N), where: x = Number of DAMs mapped to the pathway, *n* = Total number of DAMs, y = Number of all identified metabolites mapped to the pathway, N = Total number of identified metabolites. The statistical significance of pathway enrichment was evaluated using the hypergeometric test, with a hypergeometric *p* < 0.05 indicating significantly enriched pathways.

### 2.6 Preparation of prokaryotic transcriptome samples

Exponentially growing cultures (OD600 ≈ 0.8) of the *G. parasuis* parental strain (MY1902) and the isogenic Δ*qseC* mutant were harvested by centrifugation (4 °C, 4,000 rpm, 5 min) to capture transcriptomes representative of mid-logarithmic phase growth. Total RNA was isolated from the bacterial pellets using the OMEGA Bio-Tek Bacterial Total RNA Extraction Kit according to the manufacturer’s protocol to obtain high-quality, genomic DNA-free RNA suitable for sequencing. RNA integrity and concentration were rigorously assessed using an Agilent 2100 Bioanalyzer (Agilent Technologies, CA, United States) to ensure sample quality met the stringent requirements for downstream strand-specific library construction.

### 2.7 Strand-specific RNA-seq library construction and quality control

Ribosomal RNA (rRNA) was depleted from qualified total RNA samples using a prokaryote-specific rRNA removal kit to enrich messenger RNA (mRNA) and non-coding RNAs. The purified rRNA-depleted RNA was fragmented under optimized conditions. First-strand cDNA synthesis was performed using random hexamer primers. To enable strand-of-origin determination, dUTP was incorporated during second-strand cDNA synthesis instead of dTTP. The resulting double-stranded cDNA underwent end repair, 3′-end adenylation (A-tailing), and ligation of Illumina-compatible adapters. Adapter-ligated fragments within the target size range (typically 250–450 bp) were size-selected. Uracil-containing second strands were enzymatically digested using USER enzyme (NEB) to generate strand-specific libraries. The libraries were then amplified with index primers via limited-cycle PCR and purified. Final library concentration was quantified using Qubit fluorometry and qPCR, while fragment size distribution was verified using an Agilent Bioanalyzer. Libraries passing quality control (appropriate size distribution, absence of adapter dimers, sufficient concentration) were pooled in equimolar ratios based on qPCR-derived effective concentrations to achieve uniform sequencing depth across samples during paired-end sequencing (2×150 bp) on an Illumina NovaSeq 6,000 platform.

### 2.8 Bioinformatics analysis of transcriptome

The method for standardizing differences in transcriptomic data in this study is TMM. The differential analysis software is edgeR, and the filtering threshold is: padj≤0.005, |log2FoldChange| ≥1.0. Bioinformatic analysis commenced with alignment of filtered sequencing reads to the *G. parasuis* reference genome using Bowtie 2 to facilitate transcript reconstruction. Rockhopper software assembled these alignments, identifying novel transcript regions through comparison with annotated gene models; putative functions for novel transcripts were assigned via Blastx against the non-redundant (nr) database. Gene expression quantification (FPKM metric) was performed using HTSeq v0.6.1. Differential expression analysis between MY1902 and Δ*qseC* strains was conducted with DESeq2, defining differentially expressed genes (DEGs) as those with Benjamini-Hochberg adjusted *p* < 0.05 to control false discovery. Functional enrichment analysis of DEGs elucidated the biological impact of *qseC* deletion: GOseqR identified enriched Gene Ontology terms (*p* < 0.05, length-bias corrected), KOBAS revealed significant KEGG pathways, and protein–protein interaction networks were reconstructed from DEGs using the STRING database to uncover functional associations.

### 2.9 Quantitative real-time reverse transcription-PCR (qRT-PCR) validation

The qRT-PCR was performed to validate RNA-seq results by assessing transcription levels of eight randomly selected differentially expressed genes in *G. parasuis* strains MY1902 and Δ*qseC*. Bacterial cultures were harvested at mid-log phase (OD_600_ = 0.8), followed by total RNA extraction and reverse transcription into cDNA. Using 16S rRNA as the endogenous control (primers in Table 1), reactions were conducted in technical triplicates on a LightCycler^®^ 96 system (Roche) with SYBR Premix Ex Taq II (Takara Bio) under standardized cycling conditions: 95 °C/30s; 45 cycles of 95 °C/5 s, 54 °C/30s, 72 °C/30s; and melting curve analysis. Gene expression fold-changes were calculated via the 2^−^ΔΔCT method to confirm transcriptomic differences between strains.

**Table 1:** Primers used for microarray results validation with qRT-PCR.

GenBank ID	Primer	Sequence (5′–3′)	Length (bp)
A4U84_RS05770	A4U84_RS05770-F	TTATTAGCGGTTTCTACTGT	217
A4U84_RS05770	A4U84_RS05770-R	TAAACTCGCCTTTGTCG	217
A4U84_RS03670	A4U84_RS03670-F	TGGTTAGGGTGACTTCTT	283
A4U84_RS03670	A4U84_RS03670-R	GACGGACTTGAGGTGTTA	283
A4U84_RS07030	A4U84_RS07030-F	GCAAAGAAACGCTCAAAC	123
A4U84_RS07030	A4U84_RS07030-R	CAAACCAGGTGCGGAAAT	123
A4U84_RS01515	A4U84_RS01515-F	TCTGCCATCGGGAAACTA	183
A4U84_RS01515	A4U84_RS01515-R	TGGGCTTGCCTATGTGCT	183
A4U84_RS06510	A4U84_RS06510-F	TTTATTGGTGCTAGTGATTC	139
A4U84_RS06510	A4U84_RS06510-R	ATAGGCATTCGTAAGGGT	139
A4U84_RS06695	A4U84_RS06695-F	TTGCTGCTACAATGATACCT	149
A4U84_RS06695	A4U84_RS06695-R	TGAACAAAGCCAACGAAC	149
A4U84_RS02625	A4U84_RS02625-F	TGCGGTTGTTACTGTCT	286
A4U84_RS02625	A4U84_RS02625-R	CATTGCTAATCGTCCAG	286
A4U84_RS07565	A4U84_RS07565-F	TTACCGCAAGATGTGAC	152
A4U84_RS07565	A4U84_RS07565-R	GAGCCATTTATTAGACGAG	152
A4U84_02995	16SrRNA-F	CCACCTGCCATAAGATGAGC	122
A4U84_02995	16SrRNA-R	GGACCGTGTCTCAGTTCCAG	122

GenBank ID, Number of genes in NCBI GenBank database; Primer, Primer name; Sequence (5′–3′), Primer sequence; Length (bp), Gene length.

### 2.10 Combined analysis of transcriptome and metabolome

Integrated transcriptomic-metabolomic analysis was conducted to identify key post-transcriptional regulators (e.g., RNA-binding proteins, non-coding RNAs) governing metabolic flux in *G. parasuis*. This approach correlated RNA-level expression patterns with small-molecule metabolite dynamics to elucidate system-level adaptations resulting from *qseC* deletion.

### 2.11 Statistical analysis

All experimental data were analyzed using GraphPad Prism 6.0, with continuous variables assessed by Student’s *t*-test (two-group) or one-way ANOVA (multi-group), survival data analyzed via log-rank (Mantel-Cox) test, and statistical significance defined as *p* < 0.05 (denoted by asterisks).

## 3 Results

This study tested the central hypothesis that QseC, a membrane-associated histidine kinase, orchestrates critical envelope-metabolic homeostasis in *G. parasuis*. We proposed QseC regulates: (i) structural integrity through cell envelope maintenance, and (ii) metabolic flux via stress-responsive pathways. To evaluate this, we systematically analyzed Δ*qseC* mutants using multimodal approaches-initiating with ultrastructural assessment to probe envelope defects predicted for a membrane sensor, followed by metabolomic/transcriptomic profiling to identify downstream functional impacts.

### 3.1 TEM revealed QseC-dependent structural defects

**Figure 1:**
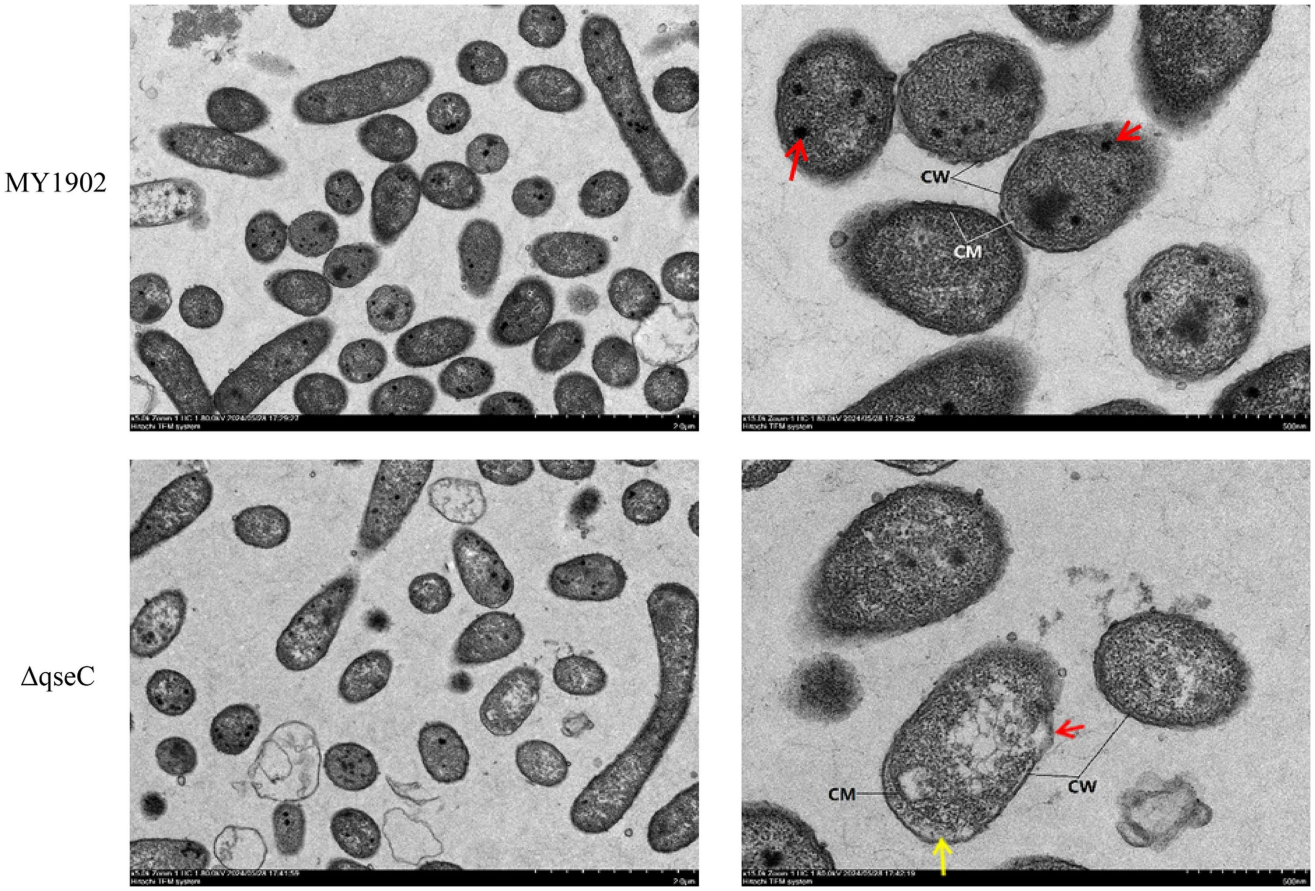
Ultrastructural comparison of *G. parasuis*. Wild-type MY1902 displayed intact envelopes and dense cytoplasm. Δ*qseC* showed wall dissolution (red arrows) and cytoplasmic detachment (yellow arrows).

As QseC is a membrane-localized sensor kinase, we first interrogated whether its deletion compromises cellular integrity using TEM-the gold standard for ultrastructural analysis. Wild-type MY1902 exhibited intact cell walls and membranes without cytoplasmic separation, with electron-dense cytoplasmic inclusions observed (Figure 1A, red arrows). In contrast, Δ*qseC* mutants showed focal wall/membrane dissolution (Figure 1B, red arrows), cytoplasmic separation (Figure 1B, yellow arrows), and protoplast disintegration. These defects indicate QseC was essential for maintaining envelope integrity and cytoplasmic organization in *G. parasuis*, validating our structural stability hypothesis.

### 3.2 Metabolomic profiling revealed QseC-dependent reprogramming

Given the structural defects observed, we next performed untargeted metabolomics to test whether QseC deletion disrupts metabolic networks supporting envelope biogenesis and stress adaptation. We performed untargeted metabolomics using UHPLC-Q-TOF MS. Data were deposited in MetaboLights (MTBLS12504).

#### 3.2.1 Metabolic alterations in Δ*qseC*

**Figure 2:**
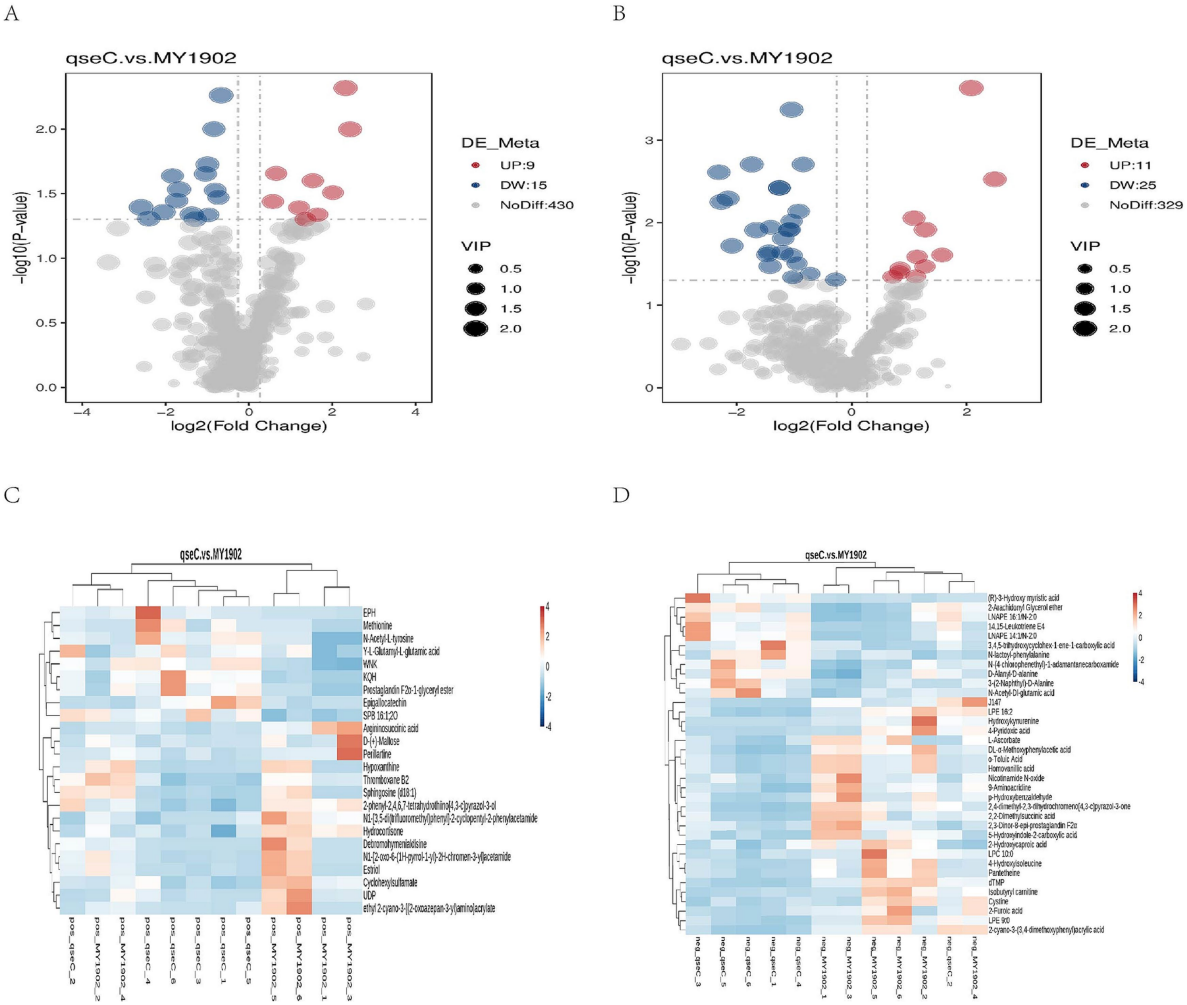
Metabolic alterations in in Δ*qseC*. **(A,B)** Volcano plots prioritized significantly altered metabolites (*p* < 0.05) in positive/negative ion modes based on fold change (log_2_[FC], horizontal axis) and statistical significance (−log_10_[*p*-value], vertical axis). Red/blue points denote up/downregulated metabolites; dot size indicates variable importance. **(C,D)** Hierarchical clustering of dysregulated metabolites revealed coordinated expression patterns functionally linked to cell envelope biosynthesis (explaining TEM-observed damage) and nucleotide metabolism. Shorter branch lengths indicate higher similarity between metabolites or samples.

We detected 819 metabolites (Supplementary Tables S1, S2), with lipids (21.89–37.00%) and organic acid derivatives (18.45–28.33%) as dominant classes. Principal component analysis confirmed analytical reproducibility through tight QC clustering (Supplementary Figures S1, S2). We investigated how deleting the *qseC* gene changes bacterial metabolism. Our comparison of Δ*qseC* and MY1902 strains found 60 significantly changed metabolites (Figures 2A,B; Supplementary Tables S3, S4). In positive ion mode, 24 metabolites differed: 9 increased (like prostaglandin F2α-ester) and 15 decreased (including nucleotide metabolites hypoxanthine and UDP). In negative ion mode, 36 metabolites changed: 11 increased (lipid signaling molecules) and 25 decreased (structural building blocks like cysteine). Volcano plots showed these changes based on statistical importance. Cluster analysis revealed connected metabolic networks (Figures 2C,D). The differentially abundant metabolites functionally explain TEM-observed structural damage and demonstrate QseC’s role as a metabolic orchestrator—depletions in envelope precursors directly correlate with membrane dissolution, while altered stress metabolites reflect compromised homeostasis. The altered metabolites explained the structural damage seen in TEM images, showing QseC controlled cellular chemistry. Lower cell wall building blocks caused membrane breakdown, while changed stress chemicals indicated lost internal balance.

#### 3.2.2 KEGG pathway analysis revealed functional impacts

**Figure 3:**
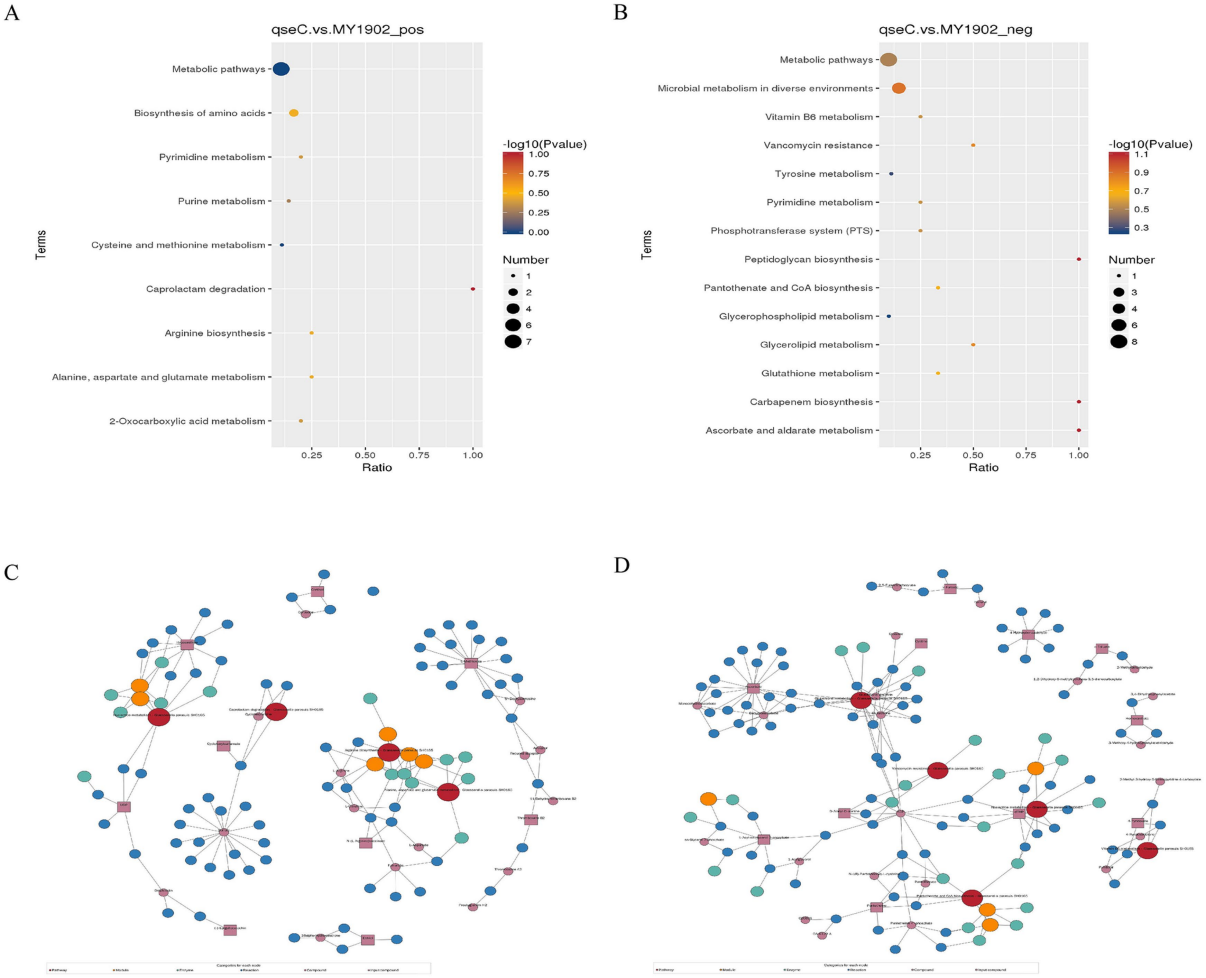
Metabolic pathway dysregulation in Δ*qseC*. **(A,B)** Bubble plots showing significantly enriched KEGG pathways (*p* < 0.05), with dot size indicating metabolite count and color indicating *p*-value significance. **(C,D)** Regulatory networks connecting altered pathways (red nodes) to Δ*qseC*-specific metabolites (purple squares), where lines denote functional relationships to cell structural defects.

To establish system-level consequences of metabolic dysregulation, we mapped differentially abundant metabolites to biological pathways. Bubble plots (Figures 3A,B) showed significant enrichment in nucleotide/amino acid metabolism (positive ion mode) and cell envelope biosynthesis and membrane function (negative ion mode). Regulatory network mapping connected these metabolic changes to cell wall damage (Figures 3C,D). Pathway analysis confirmed QseC maintained essential links between metabolism and cell structure. Its deletion disrupted the flow of building blocks for cell walls and weakened stress response capacity. This mechanistically explained the structural damage observed earlier.

### 3.3 Transcriptome analysis of Δ*qseC* mutant

Based on QseC’s predicted function as a signal transduction kinase, we hypothesized it directly regulates transcriptional programs for envelope maintenance and stress adaptation. This hypothesis was tested through comparative transcriptomics of WT and Δ*qseC* strains. The raw transcriptome data have been deposited in the National Center for Biotechnology Information (NCBI) database under the accession number PRJNA1297586.

#### 3.3.1 Sequencing data quantitation

To establish a reliable foundation for detecting transcriptional changes, we first validated RNA-seq data quality and batch effects. The Illumina HiSeq™ 2,500 platform sequenced six samples (three per strain). Wild-type MY1902 produced 14,856,120 clean reads after filtering. The Δ*qseC* mutant produced 14,932,644 clean reads. Both groups met quality standards (Q20 > 98.99%, error rate 0.01%; Supplementary Table S7). To analyze gene expression, we mapped reads using Bowtie 2. Mapping rates were high (MY1902: 92.04%, Δ*qseC*: 94.29%). These exceeded the 70% minimum requirement. Multi-mapped reads were below 10% (Supplementary Table S8). This confirmed good reference genome selection and no contamination. We quantified gene expression using FPKM values (Supplementary Table S9). Density plots showed similar expression distributions across samples (Supplementary Figure S3A). PCA analysis clearly separated wild-type and mutant groups (Supplementary Figure S3B). These results confirmed technical robustness and distinct transcriptional states in the mutant.

#### 3.3.2 Differential gene expression analysis

**Figure 4:**
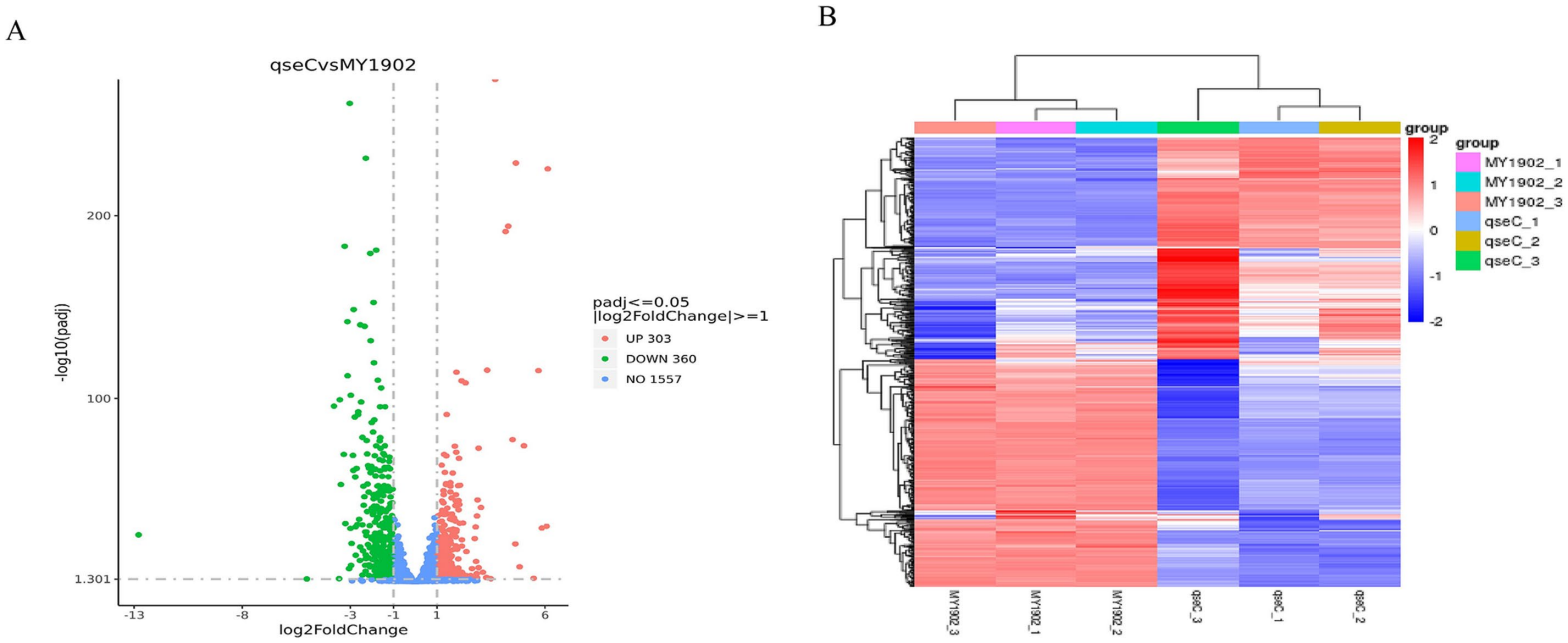
Differential gene analysis. **(A)** Volcano plot showing DEGs between wild-type and Δ*qseC* strains. Red dots: upregulated genes. Green dots: downregulated genes. **(B)** Clustered heatmap of DEG expression patterns. Red indicates high expression. Blue indicates low expression.

To identify QseC-dependent transcriptional targets, we analyzed differentially expressed genes (DEGs) using DEGseq. This revealed 663 significant DEGs (303 upregulated, 360 downregulated; Figure 4A, Supplementary Tables S10, S11). Hierarchical clustering was performed to group genes with similar expression patterns. The resulting heatmap (Figure 4B) showed distinct clusters. Genes within each cluster likely share related functions. This demonstrated that QseC broadly coordinates transcription of envelope/stress pathways.

#### 3.3.3 Functional enrichment analysis of DEGs

**Figure 5:**
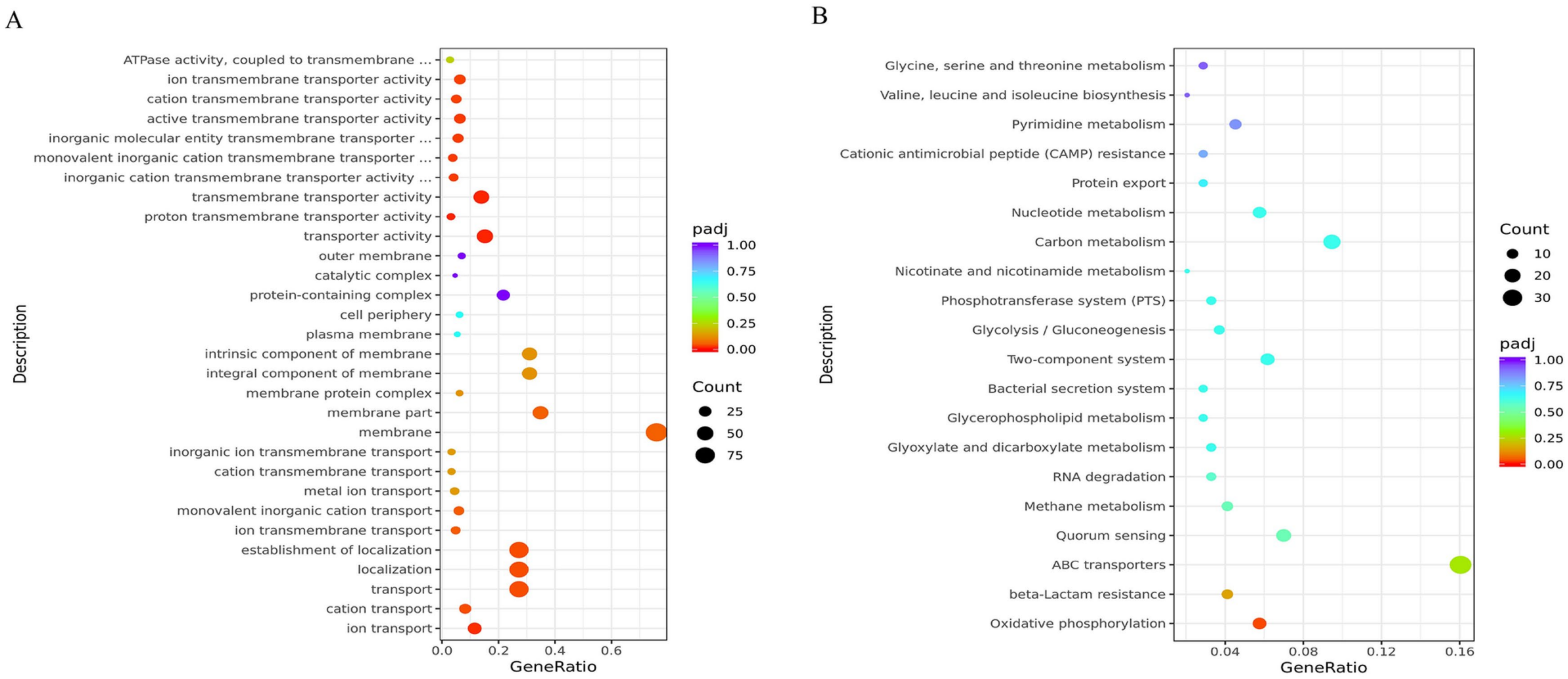
Functional enrichment analysis of differentially expressed genes. **(A)** GO term enrichment results. Dot color indicates enrichment significance (red = high). Dot size represents gene count. **(B)** KEGG pathway enrichment results. Color intensity shows enrichment strength (red = strong). Dot size indicates gene numbers. Both analyses reveal biological pathways significantly altered by *qseC* deletion.

To define biological processes controlled by QseC, we mapped DEGs to functional pathways. The 663 DEGs were assigned to 354 GO terms. Transport-related processes were most prominent, including ion transport and transmembrane transporter activity. Enrichment analysis revealed significant GO terms (Supplementary Table S12), with the top 30 shown in Figure 5A. KEGG pathway analysis mapped these DEGs to 59 pathways. Key enriched pathways included ABC transporters (39 genes), two-component systems (15 genes), and quorum sensing (17 genes). The top 20 enriched pathways are shown in Figure 5B (full results in Supplementary Table S13). These findings validated QseC as a master regulator of signal transduction and envelope homeostasis.

#### 3.3.4 Validation of RNA-seq results by qRT-PCR

**Figure 6:**
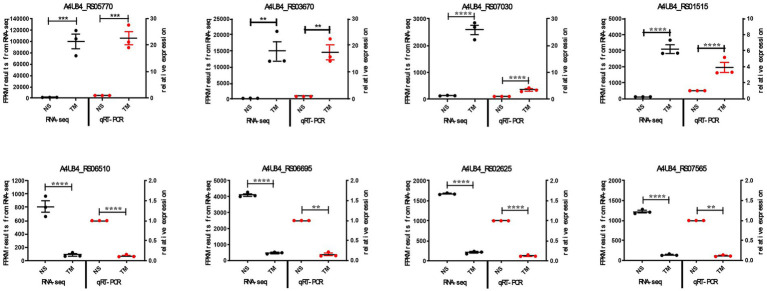
qRT-PCR validation of RNA-seq results. Expression levels of eight DEGs in wild-type vs. Δ*qseC* strains. Data show mean ± SEM. **p < 0.01, ***p < 0.001, ****p < 0.0001.

We performed qRT-PCR to confirm the reliability of our transcriptome data. Eight differentially expressed genes (four upregulated, four downregulated) were randomly selected. Using 16S rRNA as reference, we measured their expression in both strains. The qRT-PCR results matched the RNA-seq patterns for all eight genes (Figure 6). This independent validation confirmed the accuracy of our transcriptome analysis.

### 3.4 Integrated transcriptome-metabolome analysis

**Figure 7:**
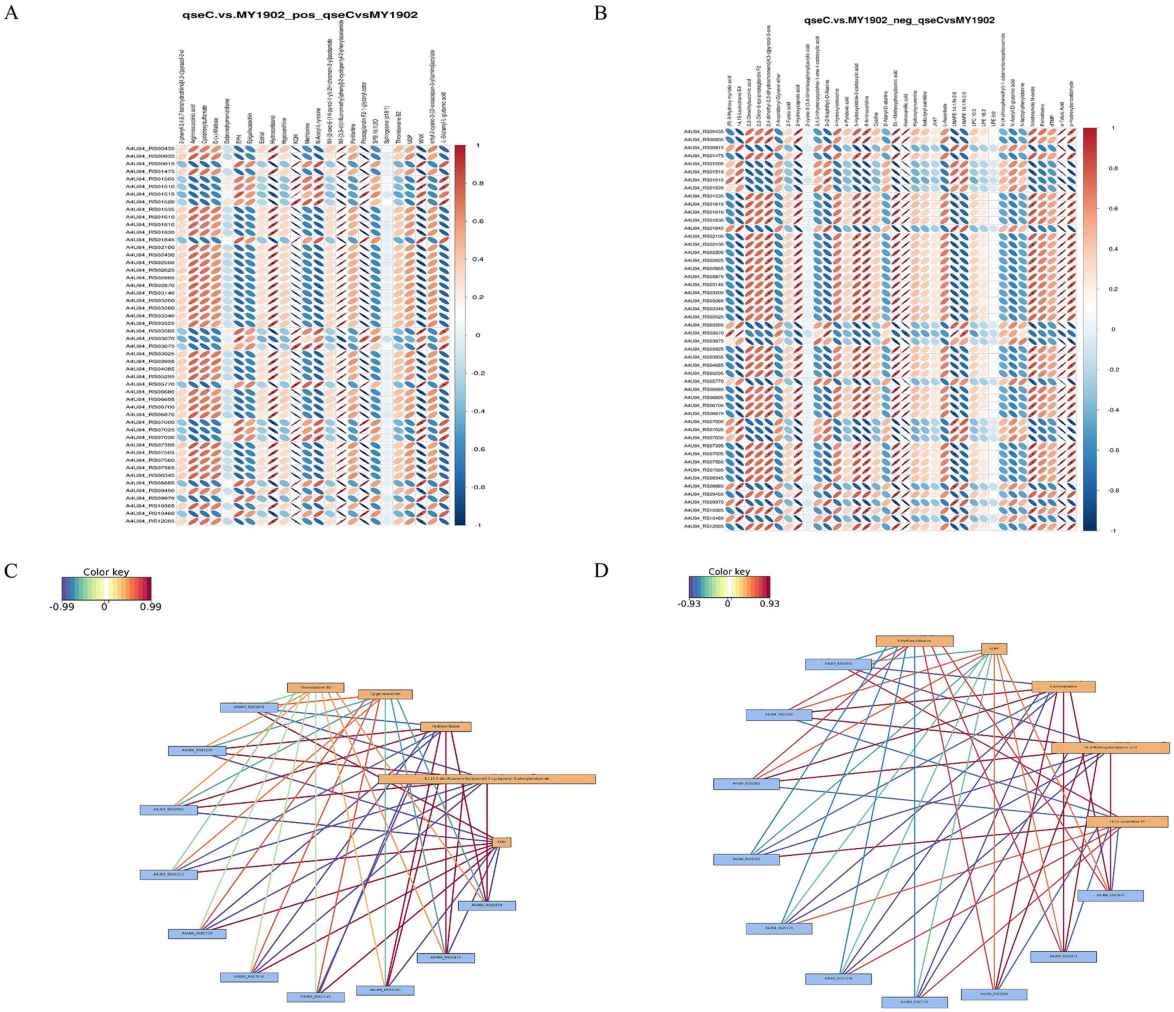
Integrated transcriptome-metabolome correlation analysis identifies *qseC*-dependent regulatory relationships. **(A,B)** Correlation heatmaps between differential metabolites and genes in **(A)** positive and **(B)** negative ion modes. Vertical axes represent metabolites; horizontal axes represent genes. Blue indicates negative correlations, red indicates positive correlations. Matrices display top 500 entities sorted by ascending *p*-value. **(C,D)** Interaction networks of key molecular relationships in **(C)** positive and **(D)** negative modes. Yellow boxes denote metabolites; blue boxes denote genes. Line colors reflect correlation strength (red indicates positive, blue indicates negative), with darker hues representing stronger correlations.

**Figure 8:**
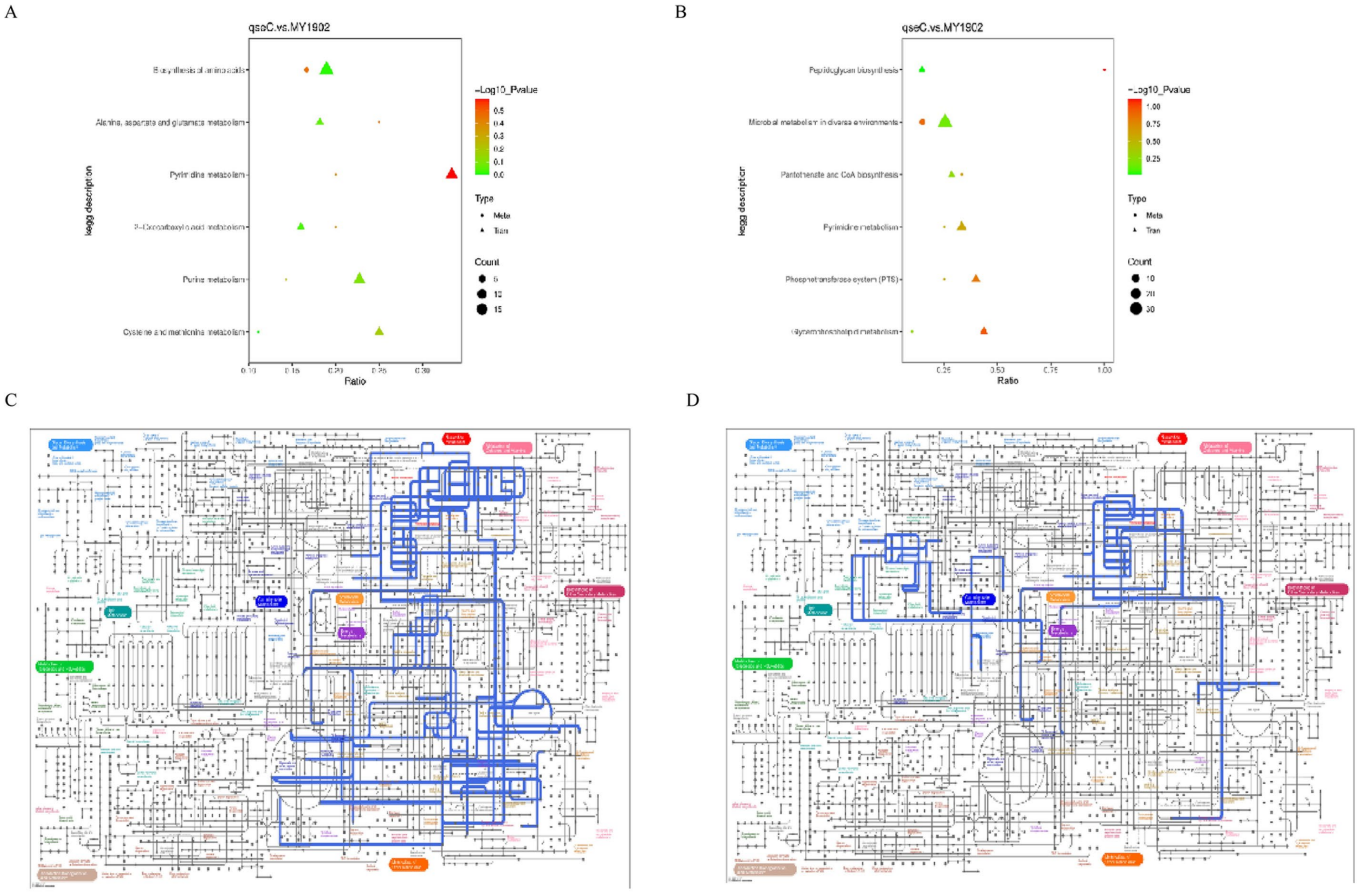
Integrated pathway analysis reveals *qseC*-dependent disruption of metabolic networks. **(A,B)** KEGG enrichment bubble plots for co-altered biomolecules in **(A)** positive and **(B)** negative ion modes. Horizontal axes represent enrichment ratio (rich factor); vertical axes list significantly enriched pathways (*p* < 0.05). Bubble size corresponds to biomolecule count; color intensity indicates statistical significance (brighter red indicates lower *p*-value). **(C,D)** IPath metabolic network mapping of consensus pathways in **(C)** positive and **(D)** negative modes. Nodes represent metabolites/enzymes; edges indicate biochemical reactions. Blue-highlighted routes denote pathways with coordinated transcriptome-metabolome alterations validated in **(A,B)**.

We integrated transcriptomic and metabolomic data to identify regulatory networks affected by *qseC* deletion. Pearson correlation analysis revealed significant associations between differentially expressed genes and metabolites. The top 50 genes and metabolites with strongest correlations were shown in Figures 7A,B. Key relationships between the top 10 DEGs and top 5 metabolites were displayed in Figures 7C,D (Supplementary Tables S14, S15). To map shared biological pathways, we analyzed KEGG enrichment. In positive ion mode, co-enriched pathways included amino acid and purine metabolism. In negative ion mode, enriched pathways involved cell wall synthesis and lipid metabolism (Figures 8A,B, Supplementary Tables S16, S17). Integrated visualizations showed coordinated changes in these pathways (Figures 8C,D). These results demonstrated that QseC synchronizes transcriptional and metabolic programs to maintain structural-metabolic coupling.

**Figure 9:**
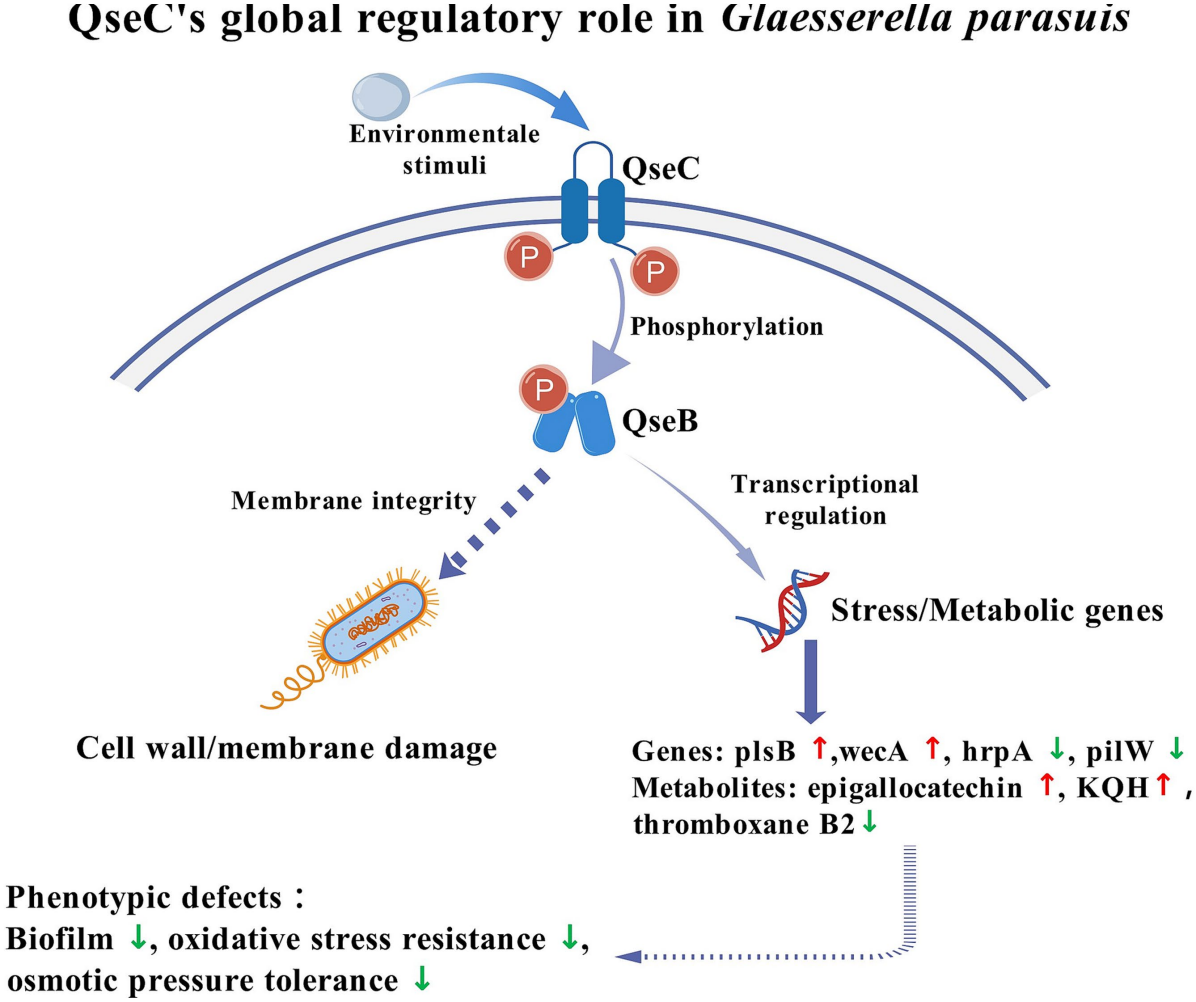
QseC mediated environmental adaptation and toxicity regulation network.

Environmental stressors activated the histidine kinase QseC, triggering phosphorylation of response regulator QseB. This phosphorylation drove transcriptional reprogramming with upregulation of membrane genes *plsB* and *wecA*, and downregulation of *hrpA* and *pilW*. Concurrent metabolic changes showed increased epigallocatechin and KQH alongside decreased thromboxane B2. Transmission electron microscopy confirmed membrane damage in Δ*qseC* mutants, linking this network to cell integrity maintenance. Loss of QseC function caused impaired biofilm formation, reduced stress resistance and compromised osmotic tolerance. Figure 9 illustrated this QseC-dependent regulatory pathway, confirming its role in bacterial adaptability through coordinated transcriptomic-metabolic control. The cell signal transduction pathway map was created with BioGDP.com (Jiang et al., 2024).

## 4 Discussion

The QseB/QseC system plays a key role in regulating virulence in Enterobacteriaceae and Pasteurellaceae (Weigel and Demuth, 2016). Research on virulence factors and pathogenic mechanisms of *G. parasuis* remains limited. In our study, natural transformation was employed to generate a double-gene knockout strain (Δ*qseBC*). Then we tested SC1401 and Δ*qseBC* with H_2_O_2_, high-concentration NaCl, and high-temperature. The results demonstrated that *qseB*/*C* deletion reduced osmotic pressure tolerance, oxidative stress resistance, and thermotolerance in *G. parasuis*, while impairing biofilm formation, serum bactericidal resistance, and antibiotic susceptibility (He et al., 2022).

Sequence alignment revealed high QseC homology across pathogenic bacteria, including *G. parasuis*, *Actinobacillus pleuropneumoniae*, *Haemophilus influenzae*, *Salmonella*, and *Escherichia coli*. Structural predictions confirmed conservation of critical amino acid residues in QseC among these species (He et al., 2018). Our findings established QseC’s involvement in stress adaptation, iron transport, and biofilm dynamics in *G. parasuis*, though its mechanistic role during host infection requires further investigation. Transmission electron microscopy revealed significant ultrastructural damage in Δ*qseC*, including localized cell wall/membrane dissolution, cytoplasmic detachment, and reduced cytoplasmic density compared to MY1902. These structural defects highlight QseC’s essential role in maintaining cellular integrity and metabolic homeostasis, potentially increasing susceptibility to environmental stressors.

Transcriptomic sequencing of MY1902 and Δ*qseC* using the Illumina platform identified 291 significantly differentially expressed genes (DEGs), comprising 107 upregulated and 184 downregulated genes. The upregulated genes in Δ*qseC* included *djlA*, *dusC*, *ushA*, *plsB*, *oppB*, *oppC*, *wecAt*, and *ruA*, etc. DjlA is a co-chaperone protein of the DnaJ family, and its overexpression enhances colanic acid capsule production in *Escherichia coli* by activating the Rcs phosphate relay system (Schachterle et al., 2018; Clarke et al., 1997). Michou et al. developed the *Escherichia coli* strain SuptoxD to optimize membrane protein production. This engineered strain addresses two critical challenges through *djlA* co-expression: (1) mitigating membrane protein overexpression toxicity, and (2) enhancing yield of properly folded membrane proteins (Michou et al., 2020). Researchers proposed a novel substrate recognition mechanism utilizing a small adapter molecule for Dus (TthDusC) in *Thermus thermophilus*. The crystal structure of *Escherichia coli* DusC (EcoDusC) comprises two domains: an N-terminus catalytic domain and a C-terminus tRNA binding domain (Chen et al., 2013). In *E. coli*, DusB and DusC may require *in vivo* cofactors for oxidation–reduction reactions, analogous to the NADH/Cbr1/Dph3 electron donor system in mcm^5^s^2^U34 biosynthesis biosynthesis (Lea-Marie et al., 2024). UshA functions as a 5′-nucleotidase, UDP-sugar hydrolase, and CDP-alcohol hydrolase, facilitating virulence factors expression and host immune evasion (Ribeiro and Cameselle, 2023). *PlsB*, a membrane-bound enzyme essential for unsaturated fatty acid biosynthesis, is extensively characterized in microbial systems. Both *Vibrio cholerae* FadR and *Escherichia coli* FadR bind to the *plsB* promoter region in *Vibrio cholerae*, with long-chain fatty acyl CoA thioesters reversing this binding (Feng and Cronan, 2011). As a *plsB* transcriptional repressor, FadR critically regulates LPS synthesis by modulating fatty acid precursor availability (Noga et al., 2020). The OppABCD complex (a type I ABC transporter) mediates low molecular weight peptide import for nutrient acquisition and host immune modulation (Yang et al., 2024). OppABCD consists of C-class substrate binding protein (SBP) OppA, transmembrane components OppB and OppC, and ATPase OppD containing two nucleotide binding domains (NBDs). In *E. coli*, five opp genes are arranged in an *oppABCDF* operon. The coding sequence of *oppA* and the early part of *oppB* have multiple promoter-like regions. This has sparked interest in whether *oppBCDF* and *oppA* can be transcribed separately. Masulis et al. demonstrated OppB’s autonomous transcriptional regulation under cationic peptide protamine exposure (Masulis et al., 2020). The phosphotransferase WecA of *Mycobacterium tuberculosis* initiates arabinogalactan biosynthesis and is considered a target of the anti-tuberculosis drug candidate CPZEN-45 (a Kapuzamycin derivative) (Huszár et al., 2017). CpxAR promotes growth, stress resistance, and virulence of *Actinobacillus pleuropneumoniae* by upregulating *wecA* transcription (Yan et al., 2020). The *truA* gene is essential for type III secretion gene expression in *Pseudomonas aeruginosa* (Ahn et al., 2004). Downregulated genes in Δ*qseC* included *hrpA*, *lptA*, *metF*, *recA*, *dnaJ* and *dnaK*, *tolA*, *pilW*, *znuA*, *fur*, and *groL.*, etc. HrpA plays a crucial role in *meningococcal* infection and neuronal cell transport, regulating the apoptosis-pyroptosis balance (Talà et al., 2022). In *Escherichia coli*, HrpA serves as a ribosome rescue factor through its DexH-box ATPase and 3′–5′ RNA helicase activities, resolving ribosome stalling caused by aberrant peptides or antibiotic effects (Campbell et al., 2024). LptA and LptC form an LPS transport complex. This complex moves LPS from the inner to outer membrane. Blocking their interaction causes membrane defects and cell death. These proteins represent therapeutic targets against Gram-negative pathogens (Dai et al., 2021). *Neisseria gonorrhoeae lptA* mutants showed heightened neutrophil susceptibility (Handing and Criss, 2015). MetF serves as an essential GroEL/ES substrate (Singh et al., 2020). Our study found that *metF* disruption increased bacterial L-lysine production from methanol (Ishikawa et al., 2008). RecD2 regulates replication restart. It inhibits RecA-mediated chain exchange through precursor filament suppression (Ramos et al., 2022). SOS pathway activation requires RecA-LexA interaction. This interaction generates signals via ATP/ssDNA-dependent oligomerization (Cory et al., 2022). In *E. coli*, DnaK collaborates with DnaJ and GrpE (To et al., 2022). These chaperones ensure correct protein folding (Bhandari and Houry, 2015). The Tol-Pal complex maintains cell envelope integrity. It contains three inner membrane proteins (TolA, TolQ, TolR), periplasmic TolB, and outer membrane Pal. TolA domains recognize division sites independently. The intermediate domain (TolAII) directs contraction site localization. TolAI recruitment requires TolQ. TolAIII recruitment needs TolB, Pal, and CpoB together (Hale et al., 2022). Tol-Pal complex maintains cell envelope integrity. It contains three inner membrane proteins (TolA, TolQ, TolR), periplasmic TolB, and outer membrane Pal (Baccelli et al., 2022). PilW enables natural transformation in *Streptococcus thermophilus*. It also facilitates T4P extrusion through ATPase DNA linkage (Yaman and Averhoff, 2021). PilW and PilV form core TfP subunits in acidophilic bacteria. These resemble *P. aeruginosa* PAO1 components (Alfaro-Saldaña et al., 2019). FUR proteins maintain prokaryotic metal homeostasis. They bind specific metals: iron (Fur), zinc (Zur), manganese (Mur), or nickel (Nur) (Steingard and Helmann, 2023). Their DNA interactions with auxiliary regulators fine-tune iron metabolism. These pathways are critical for virulence (Sevilla et al., 2021).

This study demonstrated the systematic impact of *qseC* deletion on *G. parasuis* through integrated untargeted metabolomics. In positive ion mode, methionine exhibited significant upregulation, indicating enhanced methyl donor metabolism (S-adenosylmethionine cycle) and antioxidant pathways (eglutathione synthesis) as responses to oxidative stress. Concurrent UDP precursor downregulation correlated with impaired peptidoglycan biosynthesis, consistent with QseC’s established role in maintaining cell wall integrity. Negative ion mode data revealed central lipid metabolism dysregulation, where elevated lysophosphatidylcholine species (LNAPE 14:1/N-2:0) suggested adaptive membrane remodeling. Pantetheine depletion reflected disrupted coenzyme A biosynthesis, potentially compromising energy homeostasis by restricting *β*-oxidation and acetylation-dependent secondary metabolism. This metabolic perturbation aligned with QseC’s proposed regulatory function in carbon utilization. Furthermore, cysteine downregulation implied redox imbalance, potentially activating thiol-mediated redox buffering systems to preserve intracellular reducing conditions.

Our integrated transcriptome-metabolome analysis identified key molecular relationships through expression correlation between differentially expressed genes and metabolites. The top five metabolites included: thromboxane B2 (0.99-fold downregulation), epigallocatechin (2.43-fold upregulation), hydrocortisone (0.84-fold downregulation), N1-[3,5-di(trifluoromethyl)phenyl]-2-cyclopentyl-2-phenylacetamide (0.67-fold downregulation), and KQH (2.32-fold upregulation). Corresponding top differentially expressed genes comprised: *plsB* (3.7-fold upregulation), A4U84_RS03200 (3.03-fold downregulation), *cysB* (*2.23-fold downregulation*), A4U84_RS01515 (4.67-fold upregulation), A4U84_RS05770 (6.14-fold upregulation), A4U84_RS07030 (4.3-fold upregulation), *dusC* (4.18-fold upregulation), A4U84_RS03260 (3.26-fold downregulation), *lptB* (*1.81-fold downregulation*), and *yjjG* (2.08-fold downregulation).

Correlation analysis demonstrated that the significantly upregulated gene *plsB* exhibited a positive correlation with metabolites such as epigallocatechin and KQH, suggesting its involvement in membrane lipid metabolism regulation and antioxidant defense due to *PlsB*’s established role in phosphatidic acid biosynthesis. Concurrently, the downregulation *cysB* and associated genes showed strong negative correlations with thromboxane B2 and hydrocortisone levels, indicating potential impairments in sulfur amino acid metabolism and glucocorticoid-mediated immune regulation pathways. Furthermore, the positive correlation between *dusC* and both epigallocatechin and KQH implied its functional role in purine salvage pathways and tRNA thiolation modification. These coordinated alterations in metabolic and transcriptional profiles revealed that QseC deletion compromised bacterial antioxidant capacity and immune evasion mechanisms through dysregulation of membrane integrity maintenance, redox homeostasis, and stress response systems. Collectively, these findings established novel insights into QseC’s central role in *G. parasuis* pathogenicity by orchestrating adaptive responses to environmental challenges.

## 5 Conclusion

This study comprehensively characterized the impact of *qseC* deletion on *G. parasuis* through integrated electron microscopy and multi-omics analysis. Transmission electron microscopy revealed compromised cell wall and membrane integrity in the Δ*qseC* mutant compared to MY1902, with significantly sparser cytoplasmic density. Metabolomics profiling identified 24 and 36 significantly altered metabolites in positive and negative ion modes respectively, implicating key pathways including oxidative stress response, inflammatory regulation, and membrane lipid metabolism. Transcriptomics analysis detected 663 differentially expressed genes primarily enriched in transport systems, metabolism processes, and signal transduction cascades. Integrated multi-omics correlation confirmed *qseC* deletion triggers systemic reprogramming of metabolic and transcriptional networks. These findings establish that QseC critically regulates bacterial environmental adaptation and virulence mechanisms by maintaining structural integrity and coordinating stress response pathways. This work provides a mechanistic foundation for developing novel anti-infective strategies targeting the QseC signaling pathway.

## Data Availability

The original contributions presented in the study are publicly available. This data can be found here: https://www.ebi.ac.uk/metabolights/, accession number MTBLS12504; https://www.ncbi.nlm.nih.gov/, accession number PRJNA1297586.
