# Supplementary material for: Combined transcriptome and metabolome analysis reveals the regulatory network of histidine kinase QseC in the two-component system of Glaesserella parasuis
Source: Front Microbiol. 2025 Aug 29;16:1637383. doi: 10.3389/fmicb.2025.1637383 (PMC12425974; doi:10.3389/fmicb.2025.1637383)
Supplement: Supplementary file 2 [file Supplementary_file_1.docx]

**
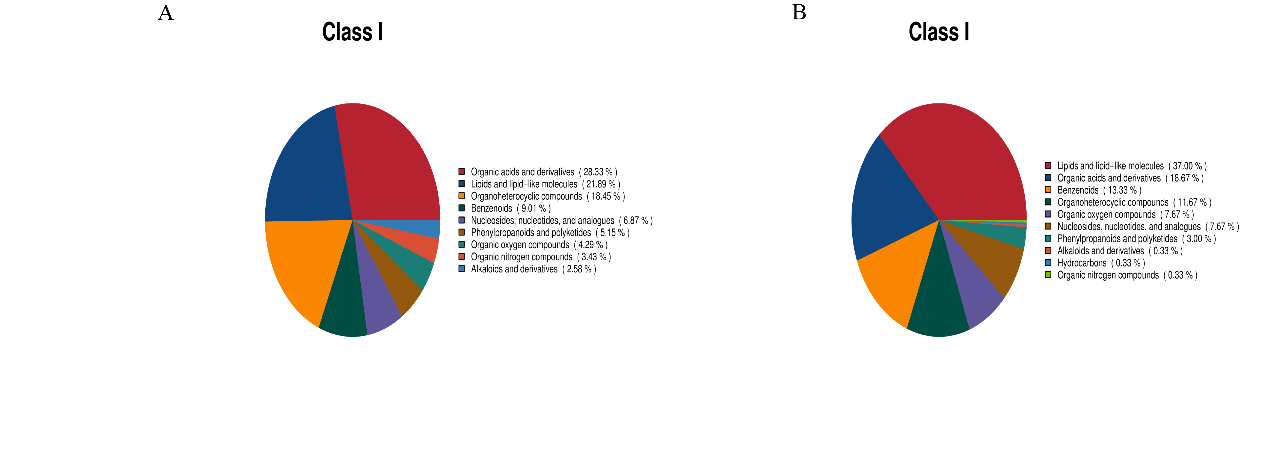
**

**Figure S1. Classification and statistics of differential metabolites in metabolomics.** (A/B) Pie chart of differential metabolite classification in positive/negative ion mode.
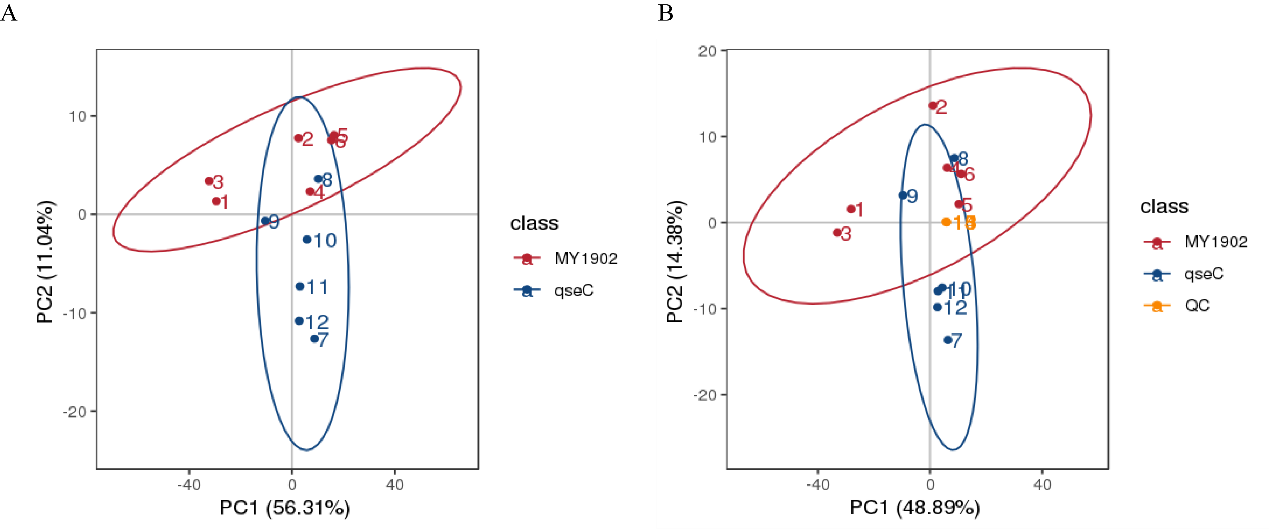


**Figure S2. PCA analysis of differential metabolite total samples in metabolomics.** PCA analysis chart of the total sample in positive/negative ion mode (A/B). The horizontal axis PC1 and vertical axis PC2 in the figure represent the scores of the first and second ranked principal components, respectively. Different colored scatter points represent samples from different experimental groups, and the ellipse is the 95% confidence interval (when the number of biological replicates is less than 4, the 95% confidence ellipse cannot be displayed).


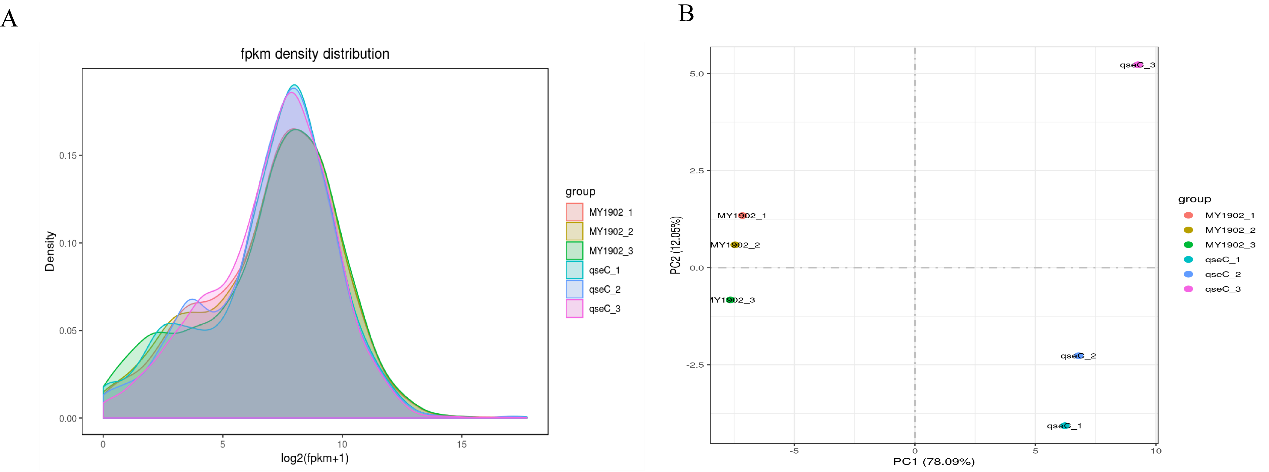


**Figure S3. Quantitative analysis of differentially expressed genes in transcriptomics.** (A) Gene expression level distribution map. The horizontal axis in the figure represents the sample name, and the vertical axis represents log2 (FPKM+1). (B) Gene expression principal component analysis chart. The horizontal axis in the figure represents the first principal component, and the vertical axis represents the second principal component.
